# The Beneficial Effects of Mesenchymal Stem Cells in Acute Kidney Injury: A Narrative Review

**DOI:** 10.2174/1574888X18666230206115046

**Published:** 2023-10-30

**Authors:** Yuxiang Liu, Jibin Han, Jingai Fang, Rongshan Li

**Affiliations:** 1 Department of Nephrology, Fifth Hospital of Shanxi Medical University (Shanxi Provincial People’s Hospital), Taiyuan, 030012, Shanxi, China;; 2 Department of the Fifth Clinical Medical College, Shanxi Medical University, Taiyuan, 030001, Shanxi, China;; 3 Department of Nephrology, First Hospital of Shanxi Medical University, Taiyuan, Taiyuan, 030012, Shanxi, China;; 4 Department of Critical Care Medicine, First Hospital of Shanxi Medical University, Taiyuan, 030012, Shanxi, China

**Keywords:** Acute kidney injury (AKI), mesenchymal stem cells (MSCs), optimization of treatment, anti-apoptosis, genetic manipulation, renal tissue

## Abstract

***Background*:** Acute kidney injury (AKI) is a multifaced disease characterized by a rapid decline in renal function. However, with growing insight into the pathophysiologic mechanisms of AKI, currently available interventions for AKI are merely supportive. Thus, novel therapies are urgently needed to improve the outcomes of patients with AKI. This narrative review aims to explore enhancing the beneficial effects of Mesenchymal Stem Cells(MSCs) in AKI.

***Methods*:** The authors examined all studies regarding the role of MSCs in AKI. And the authors undertook a structured search of bibliographic databases for peer-reviewed research literature using a focused review question. The most relevant and up-to-date research was included.

***Results and Discussion*:** Based on encouraging preclinical results, stem cell therapy has been widely explored over the last decade. Among the various stem cell types investigated, mesenchymal stem cells are being intensely investigated by virtue of their numerous strengths, such as easy derivation, undemanding cell culture conditions, anti-apoptosis, immunomodulation, and anti-inflammation effects. Mounting evidence suggests that MSCs hold great potential in accelerating kidney repair following AKI in various preclinical models. Unfortunately, low engrafting efficiency and poor survival rate of injected MSCs in the injured renal tissue are major obstacles MSCs clinical application faces.

***Conclusion*:** Various strategies, including genetic manipulation, mimicking the cellular microenvironment with different culture conditions, optimizing MSCs preparation and administration schedule, and screening patients who may more like benefit from MSCs therapy, have been developed to enhance the therapeutic potential of MSCs in AKI.

## 1 INTRODUCTION

Acute kidney injury (AKI) is a clinically heterogeneous disorder defined by a rapid increase in serum creatinine concentration with or without decreased urine output [1]. AKI can result from various aetiologies, such as major surgery, sepsis, and exposure to nephrotoxic drugs. Data from a national hospital discharge survey indicates that the occurrence of AKI in all hospitalized patients was 19.2 per 1000 hospitalizations [2]. Notably, a multinational cross-sectional study found that AKI in critically ill patients was 57.3% [3]. Patients with AKI are at high risk of short-term and long-term morbidity and mortality, posing a striking socioeconomic burden for low-income and high-income countries [4, 5].

Despite growing insight into the pathophysiologic mechanisms of AKI, few specific therapies are currently available [6]. A variety of stem cell types exhibit promising results in preclinical AKI models and are being applied in an attempt to address the rising incidence of AKI [7]. Among the various stem cell types investigated, mesenchymal stem cells (MSCs) are fibroblast-like cells with the the capacity to differentiate and potential to self-renew that have acquired remarkable attention in managing AKI [8]. The paracrine property of MSCs has made them an attractive therapeutic candidate for renal repair following AKI. Avalanche of reports from preclinical studies have demonstrated that the beneficial effects observed after MSCs administration in preclinical AKI models are mainly attributed to the secretion of the trophic factors acting in a paracrine fashion [9]. The paracrine manner of MSCs is employed *via* releasing bioactive cytokines and secreting extracellular vesicles (EVs), thereby inducing renal protective effects through anti-apoptosis, immunomodulation, anti-inflammation, and pro-angiogenesis [10, 11].

## 2 SEARCH STRATEGY

This is a narrative review. According to its nature as a “narrative”, authors chose the most relevant contributions to the matter. This article is a narrative review study and an attempt to gather information on all aspects of MSCs in AKI. The search was conducted using the keywords “acute kidney injury”in combination with “mesenchymal stem cells” in PubMed articles between 2005 and March 2022, obtaining 523 references. The authors focused on publications post-year 2005, emphasizing the past 10 years, but the authors did not exclude commonly referenced, relevant, and influential older publications. The clinical trial, case-control, review, meta-analysis studies, articles, case series, cohort, and cross-sectional studies reviewed 2005-2022. The authors also reviewed the references of each article to include further other studies or reports not identified by the search. And authors excluded articles considering the expert viewpoints and letters to the editor. Finally, a total of 97 articles were obtained and selected of 70 in analyzation.

## 3 EXPERIMENTAL STUDIES TESTING THE EFFICACY OF MSC IN DIFFERENT MODELS OF AKI

Ischemia-reperfusion (I/R) injury is a common etiology of AKI following cardiac surgery, nephrectomy or kidney transplantation. The study by Lange *et al*. demonstrated that the administration of MSCs to I/R-induced AKI in rats resulted in considerably better renal function and lower injury scores [12]. Furthermore, contrast-induced nephropathy is one of the common causes contributing to AKI and even increases the risk of developing chronic kidney disease. Peng *et al*. reported that human umbilical cord-derived MSCs markedly attenuated cisplatin-induced murine AKI by protecting mitochondria, abrogating inflammatory responses, inhibiting cell apoptosis and promoting renal cell regeneration [13]. Sepsis-associated AKI is a common life-threatening complication in critical care settings and accounts for nearly 50% of all AKI episodes. The beneficial effects of MSCs in rescuing renal functional and morphological damages have also been noted in the sepsis-induced AKI model; injecting MSCs to mice suffering cecal puncture and ligation remarkably facilitates recovery of tubular function evidenced by decreased serum creatinine and blood urea nitrogen levels [14]. The therapeutic efficacy of MSCs in attenuating kidney injury in these preclinical studies has brought tremendous interest and encouraged the initiation of clinical trials to evaluate the safety and efficacy of MSCs in patients with AKI.

## 4 CLINICAL TRIALS OF MSCS THERAPY IN PATIENTS WITH AKI

The beneficial effects of MSCs in reducing renal damage and improving kidney function in a range of different AKI models have generated considerable interest and encouraged early-phase clinical trials to assess the application of MSCs in the AKI setting. To date, 5 clinical trials have been registered on ClinicalTrial.gov to evaluate the safety and efficacy of MSCs in patients with AKI and only two clinical trials have been completed. Firstly, a phase I clinical trial (NCT00733876) with 16 cardiac surgery patients at high risk of postoperative AKI was performed to evaluate the safety and efficacy of bone marrow-derived allogeneic MSCs therapy [15]. In the study, cardiac surgery patients were treated safely with MSCs, and none of the MSC-related serious adverse events were observed during a six-month follow-up period. In addition, a phase 2 trial of 27 centers, completed in 2016, was a randomized, double-blind, placebo-controlled trial of 156 cardiac surgery subjects with early postoperative AKI aimed at determining whether MSCs reduce the time to recovery of kidney function following cardiac surgery [16]. The results of this trial also noted that MSCs infusion did not induce serious adverse events that led to treatment discontinuation. However, there were no significant differences between groups in the primary outcome. The median time to recovery of kidney function of the MSCs group and the placebo group were 15 and 12 days, respectively. Moreover, no significant differences were observed in any of the secondary outcomes, including all-cause mortality and provision of dialysis. It's worth noting that the MSC group with a longer cardiopulmonary bypass duration may be a potential reason contributing to therapy failure. Furthermore, a small subset of patients with AKI may be insufficient to detect a modest clinical effect with MSCs therapy. Clinical trials registered in the ClinicalTrials.gov database concerning MSCs therapy in patients with AKI are listed in Table **1**.

## 5 STRATEGIES TO IMPROVE THE BENEFICIAL EFFECTS OF MSCs

**Fig. (1):**
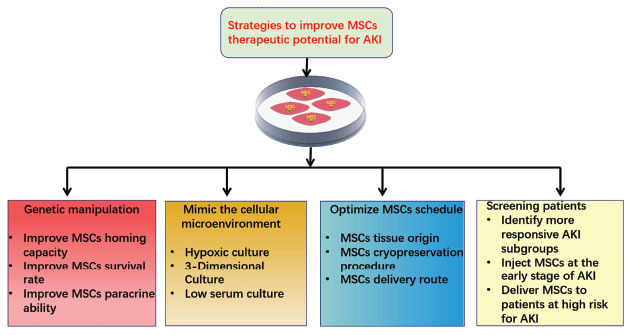
Strategies to improve the beneficial effects of MSCs, including genetic manipulation, mimic the cellular microenvironment with different culture conditions, optimize MSCs preparation and administration schedule, and screening patients who may more like benefit from MSCs therapy.

Recent clinical trials with MSCs in patients with AKI have failed to show desired therapeutic effects, thus demanding more efforts to optimize MSCs therapy in the clinical setting. Accordingly, various strategies have emerged as interesting approaches to enhance MSC potency, including genetic manipulation, mimic the cellular microenvironment with different culture conditions, optimize MSCs preparation and administration schedule, and screen patients who may more like benefit from MSCs therapy (Fig. **1**).

## 6 GENETIC MANIPULATION TO IMPROVE MSCs HOMING CAPACITY

To date, systemic administration route is still favored due to MSCs possess the intrinsic ability to home the lesion sites and colonize the damaged tissues [17]. Unfortunately, merely a small proportion of systematically transplanted MSCs finally migrate to the site of damage [18]. Low MSCs engraftment in the injured renal tissue significantly limit their therapeutic potential, thus markedly hindering their clinical application [19]. Moreover, it is well known that extensive expansion is needed to obtain a sufficient amount of MSCs for transplantation. However, cell aging and replicative exhaustion adversely affect the homing rate of MSCs.

Additionally, an avalanche of studies revealed that MSCs present low levels of homing molecules [20], which is one of the reasons that could account for the low engrafting efficiency of systematically transplanted MSCs to the target tissues. To solve the poor engraftment of transplanted MSCs, genetic manipulation has been introduced to facilitate their engraftment in AKI pre-clinical models. The stromal-derived factor-1(SDF-1)/chemokine receptor 4 (CXCR4) axis plays a crucial role in directing MSCs mobilization and facilitating MSCs adhere to the renal microvascular endothelial cell after systematic administration. The endogenous induction of SDF-1 in renal tissues is significantly increased in response to ischemic injury. Liu *et al*. showed that CXCR4-overexpressing MSCs were more effective than null-MSCs in enhancing the accumulation of MSCs in the renal tissue, along with improving the renal structure and function recovery in AKI mice [21, 22]. Moreover, transforming growth factor-β1 (TGF-β1) overexpression facilitated MSCs homing to ischemia-reperfusion injury renal tissue by upregulating expression of CXCR4 mRNA on cell membranes, thereby enhancing the therapeutic effects of MSCs in AKI treatment [23].

## 7 GENETIC MANIPULATION TO IMPROVE MSC SURVIVAL RATE

MSCs must be expanded to achieve a sufficient quantity of cells for clinical application. However, MSCs aging and replicative exhaustion adversely affected the function of transplanted cells, thereby limiting their therapeutic potential. In addition, harsh environments *in vivo* can also lead to MSCs apoptosis. Therefore, genetic manipulation has been introduced to improve the MSC survival rate in AKI by enhancing the expression of cytoprotective genes. MSCs overexpressed with heme oxygenase-1 (HO-1), an anti-apoptotic and anti-oxidative enzyme, has been shown to remarkably increase transplanted cells survival in ischemia/reperfusion-induced AKI microenvironment and correspondingly contribute to a better renal function [24, 25]. Moreover, kidney homogenate supernatant from I/R-induced AKI rat model resulted in the remarkable apoptosis of MSCs, which was reduced by erythropoietin preconditioning [26]. MSCs gene-enhanced to secrete erythropoietin has been shown to enhance tissue-protective effects of MSC in allogeneic mice with AKI, evidenced by more proliferating cells and less apoptotic cells in kidney sections [27, 28]. Kallikrein is a pleiotropic cytokine that can protect organs against oxidative damage. Hagiwara *et al.* demonstrated that genetically modified MSCs with tissue kallikrein were more resistant to oxidative stress-induced apoptosis, and administration of kallikrein-modified MSCs shown improved protection against I/R-induced AKI [29]. Nuclear factor erythroid-2 related factor 2 (Nrf2) is a vital antioxidant gene that protects various cell types against oxidative stress. In rat models of cisplatin-induced AKI, overexpression of Nrf2 in MSCs significantly promotess kidney injury recovery [30]. In line with the previous findings, Zhaleh *et al.* also demonstrated that Nrf2-overexpressed MSCs obviously reduce oxidative stress-induced injuries of glycerol-induced AKI in rat models [31]. Klotho has been recognized as a critical anti-aging protein which can suppress stem cell senescence. It has been shown that MSCs overexpressing Klotho exhibited increased proliferative capacity, enhancing the renal protective effects of MSCs [32]. Additionally, klotho-modified MSCs can further ameliorate I/R-induced AKI in rat models [33].

## 8 GENETIC MANIPULATION TO IMPROVE MSCs PARACRINE ABILITY

Accumulating evidence has emphasized that the most important mechanism of the MSC-mediated rescue renal injury relied on its paracrine capacity. Accordingly, genetic manipulation strategy has been introduced to promote MSCs to release trophic cytokines, thereby conferring additional renal protective effects. Hepatocyte growth factor (HGF) has been identified as a pluripotent factor exerting a variety of beneficial effects in tissue regeneration *via* binding the c-Met receptor. Many studies have highlighted that MSCs-derived hepatocyte growth factor (HGF) plays a vital role in the renoprotective function of MSCs by promoting angiogenesis, inhibiting fibrosis, and repairing injured tissues [34]. Remarkably, the study performed by Chen *et al*. showed that HGF overexpression in MSCs exhibited significantly better therapeutic efficacy in the I/R-induced rat AKI model [35]. Vascular endothelial growth factor (VEGF) is an angiogenic growth cytokine exerting a vital role in MSC-induced renal functional recovery [36]. It is reasonable to speculate that upregulation of VEGF in MSCs through gene transfer could promote recovery from AKI. Yuan *et al*. found that VEGF overexpression in MSCs transplantation in a mouse model of cisplatin-induced AKI conferred additional protective effects on renal function [37]. Insulin-like growth factor-1(IGF-1) is a pleiotropic cytokine with a broad spectrum of biological functions, including enhancing the blood flow and glomerular filtration rate in the kidney [38]. It's worth noting that IGF-1 is a crucial paracrine factor secreted by MSCs and participates in promoting tubular cell proliferation and silencing IGF-1 expression in MSC by small interfering RNA adversely affect the therapeutic potential of MSCs [39]. In rats with gentamicin-induced AKI, IGF-1-overexpressed MSCs further promote the amelioration of renal injury [40]. Lipocalin 2 (Lcn2), also referred to as neutrophil gelatinase-associated lipocalin (NGAL), has emerged as a critical anti-inflammatory protein involved in the proliferation and regeneration of the renal epithelial and tubular cells [41]. Roudkenar *et al*. found that genetically modified MSCs with Lcn2 enhanced protection against renal injury *via* enhancing MSCs secrete Lcn2 and an array of paracrine factors, such as HGF, IGF, and VEGF [42]. Experimental studies testing the efficacy of genetically modified MSCs in AKI models are summarized in Table **2**.

## 9 MIMIC THE CELLULAR MICROENVIRONMENT TO POTENTIATE THE BENEFICIAL ROLES OF MSCs

There is growing evidence that the local microenvironment of administered MSCs may be a predominant determinant of cell therapy outcome, and the therapeutic potential of MSCs significantly differ depending on the microenvironment at the sites of tissue damage [43]. Alternatively, the resultant cell therapy outcomes hinge on the interaction between MSCs and the inflammatory milieu. The study performed by Freytes *et al*. demonstrated that M2 macrophages and their associated anti-inflammatory cytokines may favorably affect the therapeutic outcome of MSCs, while M1 macrophage-derived pro-inflammatory cytokines adversely affect the therapeutic potential of MSCs, which may contribute to disease progression [44]. Ideally, alterations in cell culture conditions before systematic administration to mimic the hostile microenvironment present at sites of damaged renal tissues in which transplanted MSCs must function may improve therapeutic potential. This strategy presumably can enhance the capacity of MSCs to resist the harsh microenvironment, which subsequently increases their beneficial therapeutic outcome. Notably, pre-clinical AKI studies indicate that preconditioning of MSCs before transplantation, either by application of hypoxic, 3-dimensional, or low serum conditions, enhances their therapeutic ability. Experimental studies testing the efficacy of MSCs with different culture conditions in AKI models are summarized in Table **3**.

## 10 MIMIC THE CELLULAR MICROENVIRONMENT WITH HYPOXIC CULTURE

Cultivation of MSCs is commonly carried out in ambient O_2_ concentration (21%), however, the physiological oxygen concentration where MSCs usually reside in body ranges from 1% to 12% [45]. In recent years, hypoxic culture of MSCs has been investigated to mimic the normal physiological or pathological milieu *in vivo*. Notably, culturing MSCs in hypoxic conditions improved their survival in harsh environments and slowed their differentiation potential *in vitro* [46]. Hypoxic culture also enhances the paracrine secretion of cytokines by MSCs, causing upregulation of a variety of pro-angiogenic, anti-apoptotic, anti-fibrotic, and immunomodulatory factors [47]. Additionally, hypoxic culture of MSCs can upregulate both mRNA and protein levels of chemokine receptors CXCR4 and CX3CR1, which in turn enhances the capacity of MSCs to engraft into host tissues [48]. Oxygen tension plays a vital role in nearly all biological aspects of MSCs *via* modulating the hypoxia-inducible factor-1 (HIF-1) signaling pathway [49]. In general, hypoxic preconditioning can remarkably facilitate MSCs’ proliferation, migration, secretome profile, and genetic stability during *in vitro* culture, and consequently, enhance the regenerative ability of MSC-based therapy.

Owing to the above-mentioned benefits, preconditioning of MSCs with hypoxia is supposed to be an alternative option in the management of AKI. Yu *et al*. demonstrated that hypoxic preconditioning MSCs had a greater therapeutic effect on rats with ischemic AKI in contrast to normoxic preconditioning cells, mainly attributable to enhancing MSC migration and prolonging kidney retention, as determined by magnetic resonance imaging and fluorescence microscopy [50]. In agreement with previous findings, another study investigating the effect of hypoxia stimulation on MSCs demonstrated that the renal function of rats with ischemic AKI was significantly improved, principally by attenuating apoptosis and inducing angiogenesis [51]. Furthermore, preconditioning with hypoxia has been shown to enhance MSCs’ anti-fibrotic ability after administration in a rat model of ischemia-reperfusion induced AKI [52]. Hypoxic preconditioning strategy might be promising for enhancing MSCs’ therapeutic effect in the clinical treatment of AKI.

## 11 MIMIC THE CELLULAR MICROENVIRONMENT WITH 3-DIMENSIONAL CULTURE

An increasing body of evidence suggests that the properties of MSCs are contingent on the cultural environment. Thus, the strategy using 3-dimensional (3D) culture conditions has been developed to recapitulate the natural microenvironment in which MSCs would reside *in vivo*, thereby enhancing the therapeutic potential of MSCs [53]. Zhao *et al*. suggested that adipose-derived MSCs injected in a 3D aggregates form exhibited superior therapeutic effects in a rat model of I/R induced kidney injury compared with 2D cultured MSCs [54]. Furthermore, the study performed by Xu *et al*. also revealed that 3D spheroids of MSCs were more effective than cells cultured in 2D monolayer in facilitating structure and function recovery following IR-induced AKI by promoting anti-apoptotic, anti-oxidative, angiogenic, and anti-inflammatory properties [55].

## 12 MIMIC THE CELLULAR MICROENVIRONMENT WITH LOW SERUM CULTURE

Low serum culturing optimizes the paracrine effects of adipose tissue-derived MSCs for the treatment of AKI induced in nude rats by folic acid [56]. The study found that the secretion of HGF and VEGF was far preconditioning in MSCs cultured in low serum compared to that of MSCs cultured in high serum, indicating MSCs cultured in low serum possessed the great potential for tissue regeneration in AKI *via* enhancing paracrine effects.

## 13 OPTIMIZE MSCs PREPARATION AND ADMINIS-TRATION SCHEDULE

A number of studies revealed that there are substantial variabilities in MSC preparations and production. The therapeutic potential of MSCs significantly differs depending on their tissue source, cryopreservation procedure, and delivery route. Accordingly, a deep understanding of these cell processes would facilitate the therapeutic efficacy of MSCs. Indeed, both bone marrow- and adipose tissue-derived MSCs from the same donor display similar immunomodulatory effects on both innate and acquired immunity cells [57]. However, MSCs from different individuals exhibited significant donor-dependent variability in their immunological properties [58]. Therefore, HLA-matching may be carried out to minimize the humoral response between recipient and donor. Furthermore, it's worth noting that induced pluripotent stem cells (iPSCs)-derived MSCs display a more homogeneous cell population, which may favorably alter the therapeutic outcomes [59]. Cryopreservation is another MSCs preparation processing variable that is highly inconsistency across different studies. Chinnadurai *et al*. shown that cryopreservation and thawing lead to altered MSCs physiological parameters, including paracrine function, immunomodulatory activity, and proliferative and differential potential [60]. Two freezing steps are commonly applied in MSCs cryopreservation process. The study performed by Oja *et al*. demonstrated that two freezing steps do not seem to alter some important physiological properties of MSCs, but multiple freezing steps (≥4) may induce earlier senescence [61]. In addition, freshly growing MSCs may have better effectiveness as compared to thawed cryopreserved counterparts [62]. Therefore, prospective double-blinded randomized clinical studies are needed to assess the therapeutic value of both fresh and thawed MSCs. To date, there is still controversy on the best delivery route for MSCs [63]. Although the intravenous route is widely used in preclinical and clinical trials, some authors support the notion that the intra-arterial route is the optimal method to maximize the pharmaceutical potency of MSCs by delivering sufficient cells to the kidneys. Prior to MSCs transplantation, it is usually necessary to obtain a sufficient number of cells by culture expansion *in vitro*. Of note, short-term cultured MSCs are superior to long-term cultured counterparts in terms of differentiation potential, senescence status, and immunosuppressive properties [64]. As a consequence, replicative senescence may have a negative effect on the intrinsic characteristics of MSCs, which in turn attenuating the therapeutic potential. Together, it is crucial to optimize MSCs preparation and administration protocols, thereby enhancing their therapeutic properties.

## 14 SCREENING PATIENTS WHO MAY MORE LIKE BENEFIT FROM MSCs THERAPY

A growing body of evidence suggests that AKI is a heterogenous clinical entity with various etiologies and widely differing underlying pathogenesis [65]. As a consequence, screening of AKI subphenotypes which may benefit more from MSCs therapy is critical to achieving desired cell therapy outcome. Novel damage biomarkers are developed to characterize and identify these AKI subphenotypes, offering an opportunity for personalized management of AKI [66, 67]. Under ideal conditions, MSCs should be administered as soon as possible the onset of AKI. However, the principal diagnostic biomarker for AKI, serum creatinine, is not obviously elevated until renal function is severely compromised, thereby delaying MSCs therapy that might be more effective if injected earlier.

Consequently, it is reasonable to assume that functional, cellular, molecular, and genetic pathophysiological changes should be measured in order to promote earlier and more sensitive identification of AKI, allowing timely initiation of MSCs therapy. Notably, MSCs therapy has shown promising results in cardiac surgery patients at high risk of postoperative AKI. Thus, it is speculated that prompt administration of MSCs to patients at high risk for AKI within a time window may achieve a desired therapeutic outcome.

## 15 CONCERNS AND UNRESOLVED ISSUES

Although very promising preliminary results in preclinical AKI models, long-term observations and follow-up outcomes monitoring are needed to preclude the potential risk associated with these MSCs-based enhancement therapies. Notably, genetic modification of MSCs may evoke potentially serious complications offsetting its potential benefits. Furthermore, it may be unable to inject preconditioned MSCs timely following the onset of AKI due to the vast majority of preconditioning protocols being time-consuming. Continuous renal replacement therapy (CRRT) is the mainstay of therapies in critically ill patients with severe AKI. Unfortunately, MSCs injected intravenously may adhere to the surface of filter membranes through the circuit before reaching the damaged kidney tissues, impairing the therapeutic potential of MSCs as well as the efficacy of CRRT. Additionally, intravenous injection of MSCs leads to the risk of pulmonary embolism [68-70]. To date, there is limited evidence regarding the pharmacokinetics and pharmacodynamics of MSCs extracorporeally administered to patients with AKI who require CRRT. Additionally, there is still no standard regarding the isolation and culture of MSCs obtained from different tissues, making it difficult to compare different trials. The tissue used for isolation, donor age, culture media, culture methods, and long-term culture are potentially confounding variables underneath the heterogeneity of MSCs, which can lead to inconsistencies in the results of different clinical trials.

## CONCLUSION

MSC-based therapy affords huge potential for the management of AKI through secretion of paracrine factors, transfer of organelles, and cell-to-cell contact, thereby exerting anti-inflammatory, anti-oxidative stress, anti-fibrotic, anti-apoptotic, pro-angiogenic, and immunomodulatory effects. However, low engraftment and poor survival of transplanted MSCs in kidneys remarkably restrict their therapeutic effect. A variety of strategies have been explored to improve MSC therapeutic potential, including genetic manipulation, mimicking the cellular microenvironment with hypoxic or 3-dimensional (3D) culture, and optimize MSCs administration protocol. These strategies have shown promising results in preclinical AKI models.

## Figures and Tables

**Table 1:** Registered clinical trials of MSCs in patients with AKI.

**Study Title**	**Estimated Enrollment Patients**	**MSCs Origin**	**MSCs Dose**	**Primary Outcome**	**Clinical Trials. gov. Identifier**	**Status**
Mesenchymal Stem Cells In Cisplatin-Induced Acute Renal Failure In Patients With Solid Organ Cancers	9	Bone marrow derived MSCs	1X10^6^, 2X10^6^, and 5X10^6^ cells/kg	● Serum creatinine concentration	NCT01275612	Withdrawn
Clinical Trial of Mesenchymal Stem Cells in the Treatment of Severe Acute Kidney Injury	80	Umbilical cord derived MSCs	Unclear	● Serum creatinine concentration	NCT04194671	Not yet recruiting
Allogeneic Multipotent Stromal Cell Treatment for Acute Kidney Injury Following Cardiac Surgery	15	Bone marrow MSCs	1X10^6^/cells/kg	● MSC-specific Adverse Events	NCT00733876	Completed
A Study of Cell Therapy for Subjects With Acute Kidney Injury Who Are Receiving Continuous Renal Replacement Therapy	24	SBI-101*	2.5X10^8^/cells, 7.5X10^8^/cells	● Serious adverse events	NCT03015623	Active, not recruiting
A Study of Cell Therapy in COVID-19 Subjects With Acute Kidney Injury Who Are Receiving Renal Replacement Therapy	22	SBI-101*	2.5X10^8^/cells, 7.5X10^8^/cells	● Serious adverse events	NCT04445220	Recruiting

**Note:** *SBI-101 is a biologic/device combination product that combines two components: allogeneic human mesenchymal stromal cells (MSCs) and an FDA-approved plasmapheresis device. SBI-101 is administered *via* integration into a continuous renal replacement therapy circuit and is designed to regulate inflammation and promote repair of injured tissue.

**Table 2:** Experimental studies testing the efficacy of genetically modified MSCs in AKI models.

**Candidate Gene**	**AKI Preclinical Model**	**MSCs Source/Delivery Route**	**Main Findings**	**References**
CXCR4	Mice model of I/R-induced kidney injury	Mouse bone marrow/Injected into the tail vein	● Enhanced MSCs migration to the kidney ● Improved renal function, reduced acute tubular necrosis	[21, 22]
TGF-β1	Rat model of I/R-induced kidney injury	Rat bone marrow/Injected into the tail vein	● Induced homing of MSCs in repair of renal ischemic reperfusion injury	[23]
HO-1	Rats were subjected to renal I/R injury	Rat bone marrow/Injected into the tail vein	● Increased transplanted MSCs survival ● Decreased acute tubular necrosis score	[24, 25]
Erythropoietin	Mice model of cisplatin-induced kidney injury	Mouse bone marrow/Intraperitoneal injection	● Significantly less apoptotic cells and more proliferating cells	[27]
Kallikrein	Rat model of I/R-induced kidney injury	Rat bone marrow/Injected *via* the carotid artery	● Exhibited advanced protection against renal injury by suppression of apoptosis and inflammation.	[29]
Nrf2	Rat model of cisplatin-induced kidney injury	Rat bone marrow/Intravenous injection	● Inhibited apoptosis induction in MSCs transplanted into the cisplatin-induced AKI rats	[30]
Klotho	Mice model of I/R-induced kidney injury	Mice bone marrow/Injected intravenously	● Increased proliferative ability and more potent immuno-regulation ability	[32]
HGF	Rat model of I/R-induced kidney injury	Human umbilical cord/Injection through through the left carotid artery	● MSCs secreted higher levels of HGF, Promoted the amelioration of I/R-induced rat renal injury	[35]
VEGF	Mouse model of cisplatin-induced AKI	Human embryo/Intravenous tail injection	● MSCs secreted higher levels of VEGF, provided advanced benefits in the protection against AKI	[37]
IGF-1	Rat model of gentamicin-induced AKI	Human umbilical cord/Injected intravenously	● Increased the paracrine secretion of IGF-1by MSCs	[40]
Lcn2	Cisplatin-induced AKI in a rat model	Rat bone marrow/Injected intravenously	● MSCs secreted higher levels of HGF IGF, and VEGF	[42]

**Abbreviations:** AKI acute kidney injury, I/R- ischemia/reperfusion, CXCR4 chemokine receptor 4, TGF-β1 transforming growth factor-β1, HO-1 heme oxygenase‐1, Nrf2 nuclear factor erythroid-2 related factor 2, HGF hepatocyte growth factor, VEGF vascular endothelial growth factor, IGF-1 insulin-like growth factor-1, Lcn2 lipocalin 2.

**Table 3:** Experimental studies testing the efficacy of MSCs with different culture conditions in AKI models

**Culture Condition**	**AKI Preclinical Model**	**MSCs Source/Delivery Route**	**Main Findings**	**References**
Hypoxic culture	Rat model of I/R-induced kidney injury	Rat bone marrow/Intra-aortic cell delivery	● Up-regulation of CXCR4 and enhanced MSC Migration *in vitro* ● Promoted the recovery of kidney from I/R injury to a greater degree	[50]
Hypoxic culture	Rats of AKI were induced by kidney I/R surgery	Human adipose tissues /Injected into the left kidney cortex using a 28-gauge needle	● Significantly enhanced paracrine and antioxidative effects of MSCs ● Significantly improved the renal function	[51]
Hypoxic culture	Rat model of I/R-induced kidney injury	Human or rat bone marrow/Injected through the abdominal aorta	● Significantly attenuated I/R-induced renal fibrosis ● Enhanced the production of VEGF, HGF, and PGE2	[52]
3-Dimensional culture	Rats were subjected to renal I/R injury	Rat paratesticular fat /Injected into the kidney cortex using a 28-gauge needle	● MSCs are less susceptible to oxidative and hypoxia Stress ● Enhanced secretion of proangiogenic growth factors by MSCs	[54]
3-Dimensional culture	Rat model of I/R-induced kidney injury	Human adipose tissues /Injected into the kidney cortex using a 1 ml syringes	● Increased the paracrine secretion of cytokines by MSCs	[55]
Low serum culture	Rat model of folic acid-induced kidney injury	Human adipose tissues /Intravenous injection	● MSCs secreted higher levels of HGF and VEGF ● Significantly attenuated acute renal damage	[56]

**Abbreviations:** AKI acute kidney injury, I/R- ischemia/reperfusion, CXCR4 chemokine receptor 4, HGF hepatocyte growth factor, VEGF vascular endothelial growth factor, PGE2 prostaglandin E2.

## LIST OF ABBREVIATIONS

AKI: Acute Kidney Injury
CXCR4: Chemokine Receptor 4
HGF: Hepatocyte Growth Factor
HO-1: Heme Oxygenase‐1
I/R: Ischemia/Reperfusion
IGF-1: Insulin-Like Growth Factor-1
Lcn2: Lipocalin 2
NGAL: Neutrophil Gelatinase-Associated Lipocalin
Nrf2: Nuclear Factor Erythroid-2 Related Factor 2
PGE2: Prostaglandin E2
TGF-β1: Transforming Growth Factor- β1
VEGF: Vascular Endothelial Growth Factor
